# 2022 TB programme review in Pakistan: strengthening governance, with better patient diagnosis and treatment

**DOI:** 10.5588/ijtldopen.23.0587

**Published:** 2024-03-01

**Authors:** M. van den Boom, K. Bennani, C. Sismanidis, C. Gunneberg, L. Khawaja, M.A. Safdar, C. Muhwa, E. Heldal, D.M. Cirillo, A.W. Khan, R. Fatima, B.J. Khan, S. Tahseen, M.G. ElMedrek, Y. Hutin, Pakistan Joint TB Program Review Mission

**Affiliations:** ^1^ World Health Organization (WHO), Regional Office for the Eastern Mediterranean Region, Cairo, Egypt;; ^2^ WHO, Geneva, Switzerland;; ^3^ WHO, Country Office for Pakistan, Islamabad, Pakistan;; ^4^ Respiratory Society of Kenya, Kenya;; ^5^ Department of Medicine, Dermatology and Therapeutics, Kenyatta University, Nairobi, Kenya;; ^6^ Department of Infection Control and Vaccines, Norwegian Institute of Public Health, Oslo, Norway;; ^7^ Emerging Bacterial Pathogens Unit, Division of Immunology, Transplantation and Infectious Diseases, IRCCS San Raffaele Scientific Institute, Milan, Italy;; ^8^ Pakistan Ministry of National Health Services, Regulations & Coordination, Government of Pakistan, Islamabad, Pakistan

**Keywords:** tuberculosis, drug resistance, prevention/control programme, National Tuberculosis Programme

## Abstract

**BACKGROUND:**

In Pakistan, 84% of healthcare is provided by the private sector. We conducted an epidemiological and programme review for TB to document progress and guide further efforts.

**METHODS:**

Surveillance and data systems were assessed before analysing epidemiological data. We reviewed the programme at federal, provincial and peripheral levels and compiled national data along with WHO estimates to describe the evolution of epidemiological and programme indicators.

**RESULTS:**

In 2021, of the estimated number of TB cases, 55% of overall cases and 18% of drug-resistant cases were diagnosed and treated respectively. The contribution of the private sector in case detection increased from 30% in 2017 to 40% by 2021. For newly diagnosed pulmonary TB cases, the overall proportion of confirmed cases was 52%. In 2021, testing for rifampicin resistance among confirmed cases was 66% for new and 84% for previously treated patients. The treatment success rate exceeded 90% for drug susceptible TB. The main challenges identified were a funding gap (60% in 2021–2023), fragmented electronic systems for data collection and suboptimal coordination among provinces.

**CONCLUSIONS:**

The main challenges prevent further progress in controlling TB. By addressing these, Pakistan could improve coverage of interventions, including diagnosis and treatment. Bacteriological confirmation using recommended diagnostics also requires further optimisation.

## RÉSUMÉ

**CONTEXTE:**

Au Pakistan, le secteur privé assure 84% des services de santé. Une étude épidémiologique et programmatique a été réalisée sur la TB afin de recueillir des informations sur les avancées réalisées et de guider les actions à venir.

**MÉTHODES:**

Les systèmes de surveillance et de données ont été évalués préalablement à l'analyse des données épidémiologiques. Nous avons examiné le programme aux niveaux fédéral, provincial et local et compilé les données nationales ainsi que les estimations de l'OMS afin de décrire l'évolution des indicateurs épidémiologiques et du programme.

**RÉSULTATS:**

En 2021, environ 55% de l’ensemble cas de TB et 18% des cas résistants aux médicaments ont été diagnostiqués et traités respectivement. La contribution du secteur privé dans la détection des cas est passée de 30% en 2017 à 40% en 2021. La proportion totale de cas confirmés pour les nouveaux diagnostics de TB pulmonaire s'élevait à 52%. En 2021, les tests de résistance à la rifampicine parmi les cas confirmés s’élevaient à 66% pour les nouveaux patients et de 84% pour les patients déjà traités. Le taux de réussite du traitement a dépassé 90% pour la TB sensible aux médicaments. Les défis majeurs comprennent un manque de financement (60% pour la période 2021–2023), des systèmes électroniques de collecte de données fragmentés et une coordination insuffisante entre les provinces.

**CONCLUSIONS:**

Les défis majeurs entravent les avancées dans la lutte contre la TB. En les mettant en évidence, le Pakistan pourrait améliorer la portée des interventions, y compris le diagnostic et le traitement. Il est également essentiel d'optimiser la confirmation bactériologique en utilisant les diagnostics recommandés.

In 2021, WHO estimated that globally 7.9 million incident cases of TB occurred (134 cases per 100,000 population) with 1.6 million TB deaths.^1^ Pakistan (2022 population: 236 million^2^) accounts for 70% of the estimated TB incidence in the Eastern Mediterranean Region (EMR) and is therefore a regional priority. In 2011, a constitutional amendment devolved the responsibilities of the Ministry of Health to provinces, and these are now responsible for planning, implementation, monitoring, and evaluation – as well as for providing resources for health.^3^ Pakistan has a large private healthcare sector, with an estimated 5,000 private hospitals, 100,000 private general practitioners (GPs) and at least 66,000 private pharmacies. It is estimated that 85% of initial care-seeking takes place in the private sector, including 24% with informal providers and 61% with formal providers, especially GPs.^4^ In 2021, WHO estimates that more than 600,000 TB incident cases and 45,000 deaths occurred in Pakistan. Between 2019–2020, there was a drop of 17% in case notifications, which was attributed to COVID-19, followed by a 24% re-increase in 2021. Since 2005, the private sector has been involved in TB through different models of public private mix (PPM), which were extended in 2009.^5,6^ The first PPM focused on engaging private GPs, whereas the second PPM targeted health facilities of non-governmental organisations (NGOs). The third PPM deals with private hospitals. The fourth PPM considers networks of hospitals of specific organisations or agencies. A fifth PPM is being considered and will include private pharmacies.

To update previous reviews on TB in Pakistan,^7^ in 2022, an epidemiological review was conducted^8^ and a joint programme review was conducted to evaluate the progress achieved and to identify priorities for further improvement (Pakistan National TB Programme and Ministry of Health, personal communication, 2022). The first study assessed epidemiological progress, TB reporting, monitoring and evaluation systems. This informed the second study, which focused on progress and challenges of implementation in view of reaching national and international policy-relevant goals and targets. The aim of this manuscript is to summarise the findings and recommendations of these two programme reviews.

## METHODS

### Epidemiological review

We reviewed the capacity of the surveillance system to directly measure the number of TB cases and deaths according to the WHO surveillance checklist of standards and benchmarks,^9^ and analysed the epidemiological data to assess the level of, and trends in, TB, as well as the implementation of activities in the country. National health officials provided district-level data on key surveillance indicators for 2017–2021, uploaded onto a District Health Information Software 2 [DHIS2] platform with standardised analytical dashboards. This facilitated analysis and use of data according to WHO standards.^10^ Data included the number of cases notified and enrolled on treatment, according to age, gender, treatment history, laboratory confirmation, HIV status and setting (e.g., public or private and geographical location) among others.^1,11^ Data also included outcomes of treatment for disease and prevention among eligible populations. Financial data included income and sources, and funding gaps.^12^ All data presented were received from the national and provincial programmes or extracted from WHO data sources, unless otherwise explicitly mentioned. Ethical approval was not required for this study.

### Programme review

The programme review teams travelled to four provinces and the capital territory assessing, among other issues, governance, leadership and coordination, financing, the multi-sectoral accountability framework (MAF), rights and gender, supply chain management, information system, research and innovation, as well as prevention, diagnosis and treatment as recommended by WHO.^11^ National and regional documents, plans and reports were also reviewed. The programme review team interviewed stakeholders (including public and private providers, health workers, NGOs and international advisers, and met with health authorities, managers, other sectors beyond health sector (e.g., social, education and labour) and partners.

### Data analysis

The annual country data profile for Pakistan was used as a data analysis framework.^13^ Standard indicators were calculated as recommended elsewhere,^14^ including, among others, treatment coverage and outcomes, bacteriological confirmation and proportion of rifampicin-resistant TB (RR-TB) cases, stratified by new and retreatment cases, case distribution by age groups, and province of birth. Data were reported in terms of input and structure, output, outcome and impact, and discussed in terms of management and funding, diagnosis, treatment, and prevention.

## RESULTS

### Input and structure

*Financing:* In 2021, of the estimated National Strategic Plan cost of USD144 million, USD90 million funding was available (funding gap: 38% of estimated costs). Contribution included domestic sources (USD35 million, 24%) and official donor assistance (USD55 million, 38%).

*Diagnostic devices:* In 2022, there were 404 Xpert MTB/RIF sites (372 in the national programme and 32 in the private sector) for 2,678 basic management units for TB in all provinces, a 10-fold increase from 40 sites in 2015.

*Health information system:* Since 2018, Pakistan has used a District Health Information Software [DHIS2] system. Since 2021, attempts have been made to transition from the initial aggregated data to case-based data, and aggregated data from the private sector have also been included. The programme has made progress, establishing a national capacity to roll out and manage these systems, and routinely analyse and use the data produced. However, there are some data discrepancies (e.g., drug-resistant cases were entered individually in an electronic national recording system, which was still not integrated to DHIS2).

*Planning and coordination:* The national programme and its five provincial counterparts conducted their planning jointly, but with less coordination during implementation. The programme reached out to partners for the MAF-TB. WHO supported a national baseline assessment, which led to pilot implementation in Punjab and Sindh. The MAF-TB aimed at patient-centred services, early case-finding and improved treatment outcomes. In 2022, there was limited coordination across levels with no staff assigned to coordinate key areas (e.g., PPM, child TB and TB-HIV). The provincial and district levels coordination meetings had been interrupted as funding from Global Fund had been discontinued. From 2023 onwards, this mechanism has been replaced with the introduction of the post of District TB Officer (DTO), with responsibility for validating data entry after the implementation of case-based DHIS2. There were no estimates for the required human resources needs to coordinate and drive the TB response.

### Output

**Figure 1.**
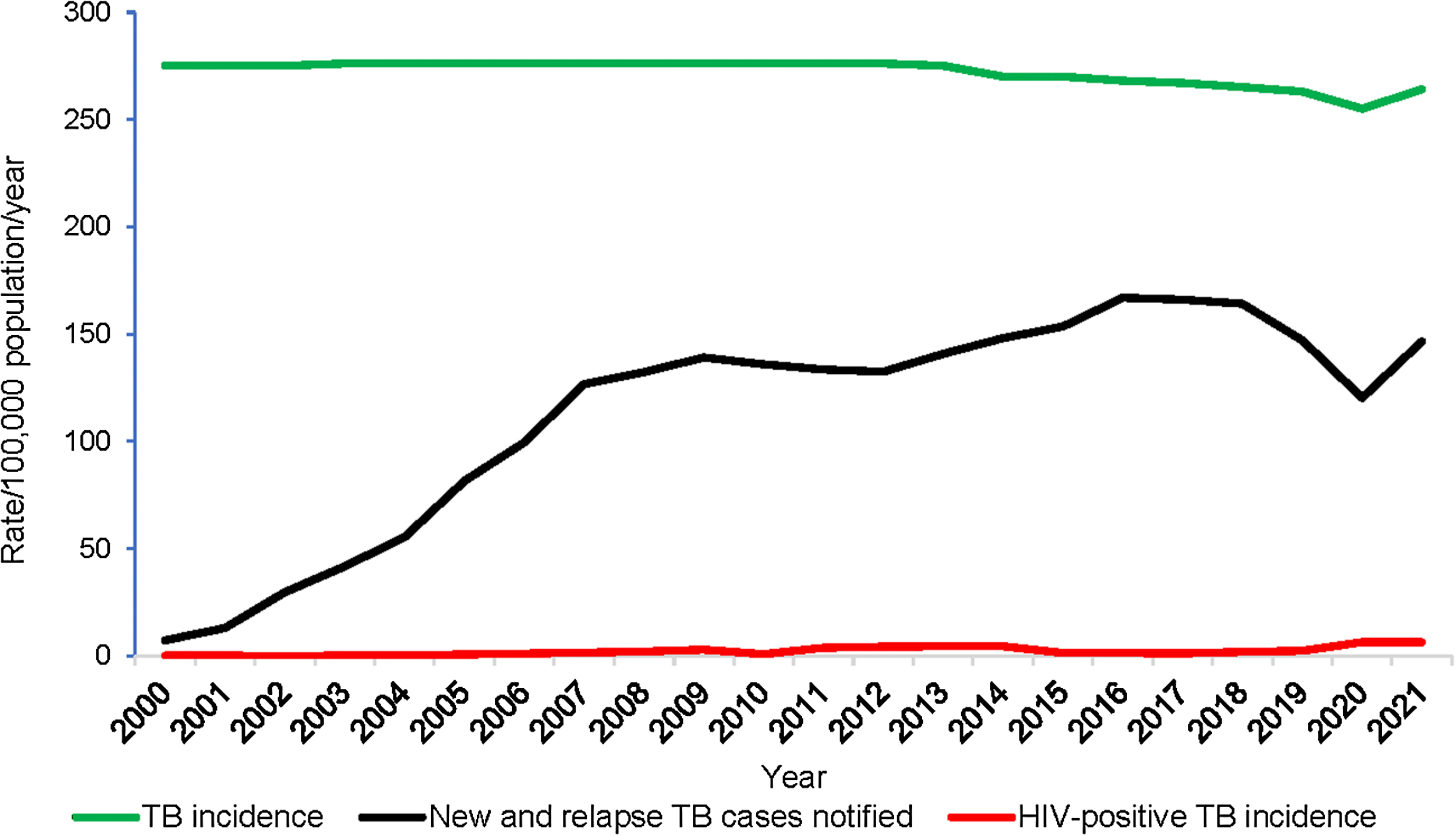
TB incidence disaggregated by HIV status and case notification rates, Pakistan, 2000–2021.

**Figure 2.**
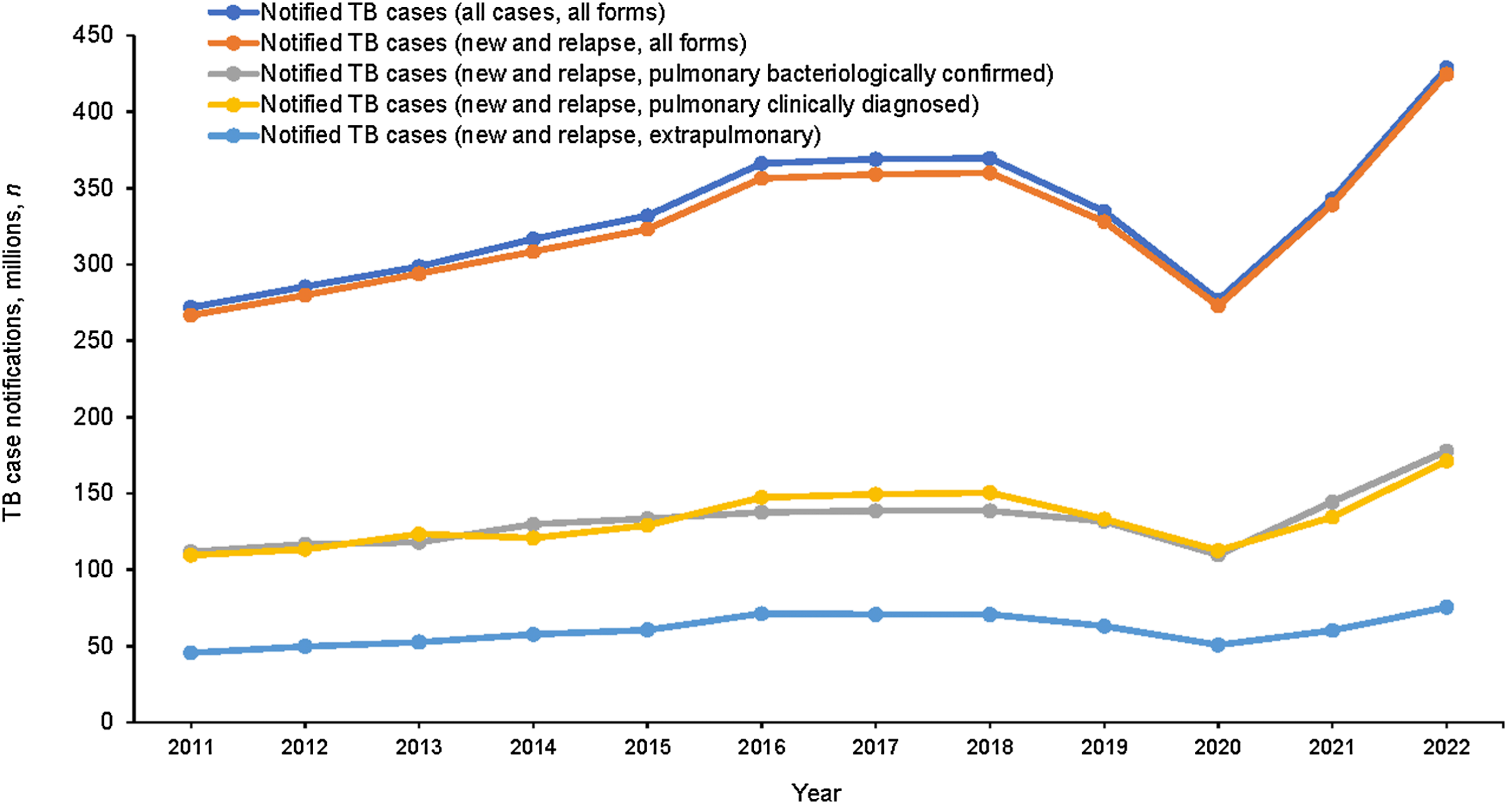
Number of TB case notifications by type, Pakistan, 2011–2022.

**Figure 3.**
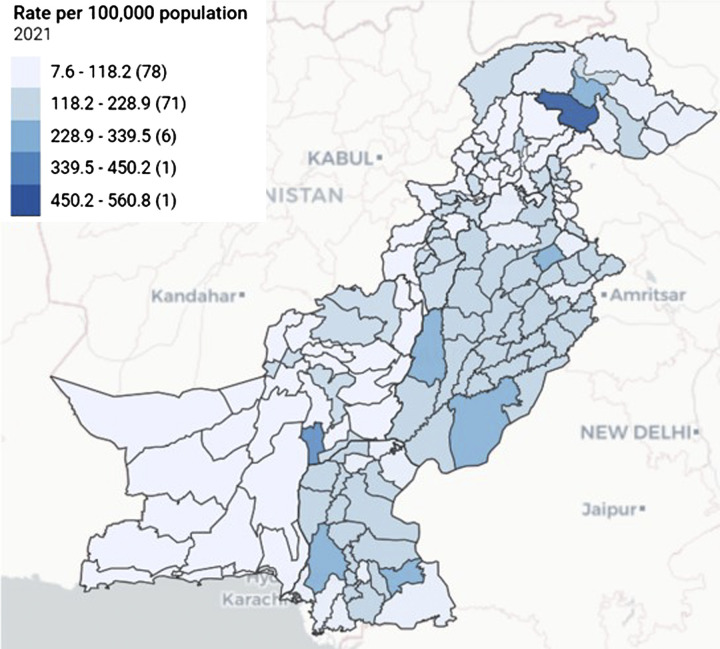
District-level TB notification rate/100,000,* all forms, 2021. *Numbers of districts in parentheses.

*Case-finding:* The number of notifications declined between 2018 and 2019, further dipped in 2020 during the COVID-19 pandemic and recovered in 2021 to reach the 2019 level (Figure 1). In 2021, 82% of notified cases were pulmonary TB (pTB). Of those, 52% were bacteriologically confirmed (Figure 2). The number of bacteriologically confirmed and clinically diagnosed pTB cases did not vary between 2017–2021, during which time the number of bacteriologically confirmed cases slightly exceeded (145,027) the number of those clinically diagnosed (134,938, Figure 2). Notification-specific geographical areas were usually proportional to the population size (e.g., in 2021, Punjab, which represents 60% of the Pakistan population, contributed to 61% of notifications). The five districts reporting the largest number of notifications in 2021 were Lahore, Faisalabad, Multan, Gujranwala and Rawalpindi, all of which (except Multan District) were the most populated districts (Figure 3). Only 18% of the estimated people with RR/multidrug-resistant (MDR)-TB (2,300) were diagnosed and treated in 2021.

*Treatment:* Guidelines were not fully capturing the latest WHO recommendations (e.g., treatment of isoniazid-resistant TB, use of shorter regimens for drug-susceptible TB [DS-TB]). There were no single formulations of first-line drugs for both adults and children, e.g., for the management of patients with adverse reaction to TB drugs. Direct observation of treatment was carried out mainly by family members or supporters with minimal use of digital adherence technologies. No approach was available to provide care post-TB including, for post-TB lung disease (PTLD).

**Figure 4.**
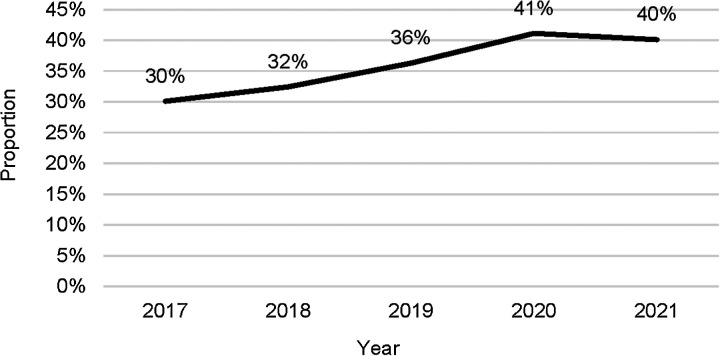
Contribution of the private sector to TB case notifications, Pakistan, 2017–2021.

*Role of the private sector:* The private sector contribution to case notifications increased from 30% in 2017 to 40% in 2021 through the various PPM models (Figure 4). In 2021, among private sector notifications, GPs accounted for 73%, followed by NGO facilities (14%), private hospitals (11%) and specific organisations and agencies (2%).

### Outcome

**Figure 5.**
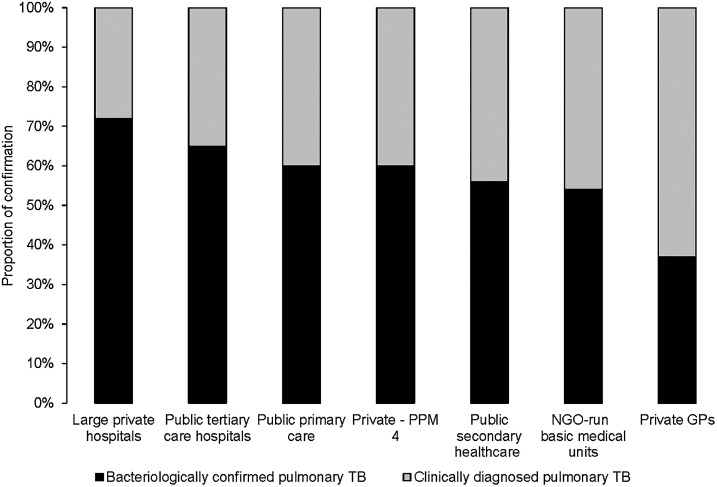
Bacteriological confirmation of TB by type of private and public sector facilities, Pakistan, 2021. PPM4 = networks of hospitals of specific organisations or agencies; NGO = non-governmental organisation; GP = general physician.

*TB diagnosis:* The proportion of pTB cases bacteriologically confirmed increased from 48% in 2016 to 52% in 2021. In 2021, among new and relapse patients diagnosed, 55% were tested with Xpert MTB/RIF. In addition, 66% and 84% of bacteriologically confirmed new pulmonary and previously treated pulmonary cases, respectively, were tested for RR. Within the public sector, laboratory confirmation of pulmonary cases reached 60% in primary facilities, 56% in secondary hospitals and 65% in large tertiary care hospitals (Figure 5). Within the private sector, the proportion of confirmation was 37% in private GP facilities, 54% in the NGO model, 60% in hospitals of specific organisations or agencies, and 72% in large private hospitals. Overall, the public sector had a higher proportion of confirmation (60–65%, median: 62.5%) than the private sector (37–60%, median: 48.5%).

*Children and contact investigations:* Between 2017–2021, the proportion of new and relapse cases notified among children under 15 years of age was 11%, with variations across provinces and regions. The national programme called for contact investigations. However, there were no explicit guidelines and sub-optimal implementation. Household contacts were requested to report for screening rather than active household visits being conducted to screen contacts.

*Treatment coverage:* The national programme notified 339,256 cases (coverage of 55%, 45% missed). The proportion of presumptive patients identified and those diagnosed with TB did not reach the level expected from the number of attendees in outpatient departments under the conservative expected proportion of those with cough (10%) and the expected prevalence of TB among those presenting with cough (10%). For example, in Punjab it was 13% in the first two quarters 2022. By 2020, 2.4% of the 5,202 Basic Health Units that constituted the first level of primary health care were engaged in TB activities. The majority of the RR/MDR-TB cases diagnosed (*n* = 2,040, 89%) were enrolled on treatment.

*Treatment outcomes:* Over the past 5 years, treatment success rates among new and relapse DS-TB cases have remained above the global target of 90% (94% for the 2020 cohort) and above the global average for MDR-TB (73% achieved for the 2019 cohort). Treatment success was lower for HIV infected TB patients (82%), retreatment patients (84%) and those with pre- or extensively drug-resistant TB (67%).

*TB and HIV:* The HIV epidemic was concentrated in high-risk groups (WHO/Joint United Nations Programme on HIV/AIDS [UNAIDS]: 210,000 infections, prevalence: 0.2% in general population) and WHO estimated that less than 5% of TB cases were associated with HIV (NTP communication). The number of TB patients who were tested for HIV increased from 10,423 in 2012 (4%) to 175,872 in 2021 (52%). In 2021, less than 1% of TB patients were HIV-positive and only 63% of co-infected TB/HIV patients had received antiretroviral treatment.

*TB drug resistance:* In 2021, WHO estimated that the overall prevalence of MDR-TB, including RR-TB, was 2.5% in new patients and 4.9% in previously treated patients (estimated number of incident MDR/RR-TB cases: 16,000, 95% confidence intervals [CI] 11,000–21,000). In the same year, 3,138 were laboratory confirmed and 2,878 (91%) were treated (18% of the estimated MDR/RR-TB cases). Xpert^®^ MTB/RIF (Cepheid, Sunnyvale, CA, USA) testing capacity was available in all 33 sites implementing programmatic management of drug-resistant TB. Among MDR/RR-TB patients, the levels of resistance to second-line drugs reached 40% for fluoroquinolones and 5% for bedaquiline, which has only been used since 2016 in Pakistan.

*TB prevention:* In 2021, the coverage of preventive TB treatment was 3% among child household contacts of contagious TB cases. No coverage estimate was available for people living with HIV. TB infection prevention and control (TB-IPC) was identified as a national emergency: in most health facilities visited, there were no written IPC facility plans with verbal reports of a high rate of TB among health care workers (HCWs) in the absence of regular programmes for their periodic screening.

*Impact:* In 2021, WHO estimated that 611,000 new TB cases occurred (95% CI 445,000–803,000; incidence rate: 264/100,000, 95% CI 192–347). The trend of incidence between 2000 and 2012 was unknown due to the lack of robust national-level data. In 2012, a prevalence survey carried out from 2010–2011 provided a prevalence estimate that allowed modelling incidence. The estimated TB incidence rate declined between 2015 and 2020 and increased in 2021. Overall, there was a 2.2% decline between 2015 and 2021. In 2021, WHO estimated the TB mortality rate at 21/100,000 (95% CI 16–25), with 48,000 deaths in HIV-negative TB cases. Despite a 1.8% increase in the number of TB deaths between 2015 and 2021, the mortality rate decreased by almost 1% per year between 2015 and 2021. The Table provides an overview of the critical indicators from input to impact.

**Table.** Critical indicators for inputs, outputs, outcomes and impact, Pakistan TB Programme, 2021.

	Islamabad Capital Territory	Punjab	Sindh	Baluchistan	Khyber Pakhtunkhwa	Pakistan
Inputs						
Annual funding, USD million*	—	7,518,419^†^	7,800,999	2,835,941	4,288,918	—
Funding gap, USD million	—	377,695,707.14	124,776,665.79	27,236,619	48,906,297.97	—
Full-time staff, *n*	—	317	522	125	—	—
Donor dependency for human resources	Yes	Yes	Yes	Yes	Yes	Yes
Laboratory SOPs and manual	Yes	Yes	Yes	Yes	Yes	Yes
Implementation of TB diagnostic algorithm	Yes	Yes	Yes	Yes	Yes	Yes
Culture laboratories/10,000,000	0	0.5	1.05	0.8	—	0.6
Microscopy laboratories/100,000	0.7	0.7	0.9	1.1	—	0.8
GeneXpert machines/100,000	0.3	0.1	0.2	0.3	—	0.2
Proportion of primary health facilities that diagnose TB, %	43	65	72	57	45	62
Proportion of secondary health facilities that diagnose TB, %	29	29	25	37	51	33
Proportion of tertiary health facilities that diagnose TB, %	29	6	3	6	4	5
Specimen referral systems	No	Yes	Yes	No	No	Variable
Training of TB staff including laboratory^‡^	Yes	Yes	Yes	Yes	No	Variable
Electronic TB information system	Yes	Yes	Yes	Yes	Yes	Yes
Outputs						
Cases reported by private sector, %	39	42	50	29	44	43
DS-TB diagnosed using GeneXpert, %	—	60	50	31	—	55
Pulmonary TB bacteriologically confirmed, %	53	51	56	52	42	51
TB cases with documented HIV status, %	23	87	35	10	6	61
New TB patients tested for RR-TB, %	—	—	—	—	—	92
Previously treated patients tested for RR-TB, %						84
TB screening of high-risk groups^¶^	—	Partial	Partial	No	—	—
Proportion of TB patients aged 0–14 years, %	7	8	17	18	34	14
Decentralised DR-TB services	—	Yes	Yes	No	—	Variable
Shorter oral treatment regimens for DR-TB	Yes	Yes	Yes	Yes	Yes	Yes
Proportion of eligible children under 5 on TPT, %	0	3-	12	1	0	5
Proportion of eligible PLHIV on TPT, %	0	2	57	35	1	13-
MAF-TB implementation	—	Yes	No	No	—	—
Operational research capacity	—	Yes	Yes	Yes	—	Variable
Outcomes, %						
TB treatment coverage	—	66	—	34	—	55
Success rate of DS-TB	84	94	94	91	96	94^†^
Success rate of DR-TB	—	73	71	53	—	73^#^
Impact						
Mortality/100,000	—	2	2	1.3	—	21
Incidence/100,000	—	196	264	264	—	264

* Domestic and international.

^†^ 2020.

^‡^ Technical competence. – DST, algorithm.

^¶^ Contacts, patient with diabetes and other non-communicable diseases, prisoners, etc.;

^#^ 2019.

SOP = standard operating procedures; DS-TB = drug-susceptible TB; RR-TB = rifampicin-resistant TB; DR-TB = drug-resistant TB; TPT = TB preventive treatment; PLHIV = people living with HIV; MAF-TB = TB multisectoral accountability framework.

## DISCUSSION

Although coverage of TB services increased in Pakistan, with 55% of estimated cases being diagnosed and treated in 2021, much work needs to be done to reach the 90% WHO targets for 2025.^1^

Similarly, the national programme was only able to identify 18% of the estimated people with MDR-TB in 2021 because of the low bacteriological confirmation of pTB (52% in 2021) and limited Xpert testing of new and relapse TB patients (55% in 2021). Several reasons may explain these gaps. First, the number of presumptive TB patients identified and screened by health services (including health workers and outpatient departments) remains insufficient. In the field during the review, insufficient screening of high-risk groups was observed, with limited active case-finding and insufficient contact-tracing. Second, TB services, including laboratory testing, were not always sufficiently available at local levels of health service provision. Third, while the proportion of TB cases notified from the private sector reached 40% in 2021, it remained well below the proportion of primary care provided in the private sector (84% in 2017). Other TB services also suffered from insufficient coverage. Preventive treatment did not reach the target of 50% of eligible persons. Identified obstacles to higher coverage included prescriber hesitancy through fear of medicines toxicity, perceived risk of creation of drug resistance and the costs of diagnostics to rule out TB disease.^15^

Stronger governance and technical management for the TB programme could address some of these gaps. At the level of input, in 2021, there was a 60% financing gap with remaining dependency on external financing that accounted for 92% of programme costs. The lack of funding could in part explain challenges in governance and management of the TB programme. From a programme management standpoint, coordination between the various levels, federal and provincial, remained incomplete. Between April 2021–October 2022, there were six leadership changes at the central level of the Central Management Unit. District and provincial coordination meetings were crucial to guide action on the basis of routine data and to monitor implementation at all levels. However, funding for these had been discontinued and the activity stopped. In terms of TB care enabling community systems, some initiatives focused on community rights and gender-based responses, but these remained insufficient. MAF, which was successfully piloted in Punjab, needs to be expanded at the national level. Finally, the TB data system remained separate from most other data systems, including, occasionally, duplication of parallel data systems within the TB programme (e.g., for DR-TB). These challenges from a strategic information point of view prevented the real-time maintenance of a monitoring and evaluation framework covering key input, process, output and outcome indicators.

In Pakistan, confirmation with WHO-recommended diagnostics (WRD) increased from 22% in 2018 to 55% in 2021, while WHO set a 100% target by 2025.^1^ Confirmation with WRD for first-line drugs was heterogeneous, and lower at the peripheral health level, including in basic management units and primary health care facilities. This underuse has consequences. Microscopy is still widely used as an initial test, leading to different algorithms being implemented at different levels and to some people with TB being missed. Insufficient diagnostic accuracy leads to false-positive and false-negative results and a failure to diagnose drug resistance leads to the prescription of inadequate regimens.^16,17^

The availability of Xpert increased from 286 in 2018 to 497 in 2022. However, the increase of diagnosis with WRD did not increase proportionately. A number of factors may explain this gap. First, availability of Xpert is high at tertiary and secondary level but insufficient in primary care, where it is most needed. Second, WHO recommendations are relatively recent. Pakistan made efforts to adapt but needs more time to procure equipment and cartridges, adapt algorithms and train staff. Third, cartridges need to be funded, requested and distributed. Fourth, insufficient linkages with, and sub-optimal specimen transportation between, primary health care facilities providing TB care and Xpert sites have been observed. Other PCR diagnostics that could also be used for TB were made available through different mechanisms (e.g., emergency and humanitarian aid) also, taking advantage of the COVID-19 pandemic. This equipment will be installed in laboratories at the divisional level with the purpose of further optimising integrated diagnosis of TB, HIV and hepatitis. All these elements suggest that Pakistan and its TB programme would benefit from an overall assessment of the molecular diagnostic pathway that could re-distribute equipment and optimise diagnosis for all communicable diseases, with a particular focus on the private sector to increase cross-programmatic efficiency. Diagnosis with WRD was lower in the private sector, particularly among GPs as 1) only 6% of GPs are involved and reporting in the national TB programme; 2) only 7% of WRD machines are in the private sector (even though the private sector accounts for 41% of case notifications); and 3) the specimen referral system is not fully linking care in the private sector with diagnosis in the public sector. Increasing the availability of WRD in the private sector and improving specimen referral could provide possible solutions.

Although treatment success is consistently high for DS patients and reasonably high for RR/MDR-TB patients, the results achieved do not cover the patients not diagnosed or diagnosed and not treated in the public sector, as well as the patients managed by the private sector. Quality treatment must be ensured for all cases based on updated guidelines, as sub-standard treatment (in terms of regimens, duration, drug quality, or adherence etc.) may lead to selection for additional drug resistance^18–20^). In Pakistan, the extensive use of fluoroquinolones for diseases other than TB (largely in the private sector) has led to an over-40% prevalence of resistance to this drug, and resistance to bedaquiline is also increasing.^21^ Furthermore, the programme review identified the importance of detecting and managing PTLD, proposing this as an operational research activity to be expanded in future.^22–24^ After having successfully completed treatment, 50–90% of TB patients suffer severe exercise limitations with hampered quality of life. The relevance of early identification of these patients and their correct management is becoming more and more important, particularly as PTLD often co-exists with long COVID.^25,26^

The programme review also recommended the expansion of provision for TB preventive treatment (TPT) as currently recommended by the National TB Programme (NTP). The priority is children below the age of 5 who are household contacts of infectious TB cases and HIV co-infected individuals, followed by household contacts of any age, while establishing a monitoring system for the preventive treatment cascade. Also, given the national importance of TB-IPC, the Programme Review recommended as a as a major priority training on TB-IPC and the development and implementation of facility IPC plans nationwide.

Our manuscript has several limitations. First, it was not possible to describe the provision of TB services in the private sector that is not engaged in the existing PPM schemes. Therefore, practitioners in the private sector may be diagnosing and treating TB without reporting it to the national programme. Contributions from the informal sector are impossible to quantify.^27^ One way of addressing this limitation might be to investigate TB medicines sales connected to prescriptions given in the private sector. Assuming there may be underreporting from the private sector, the real TB treatment coverage could exceed the estimated 55%. Second, the review did not cover the other laboratory pathways (e.g., HIV) or TB services offered as part of other programmatic activity that could be integrated with TB. These need to be examined jointly to propose arrangements that would be more efficient. Third, some of the data in terms of key indicators, such as the proportion of primary, secondary, and tertiary facilities that diagnose TB, were missed. This absence points to the need to a standardised monitoring and evaluation framework that would strengthen the accountability of the programme.

## CONCLUSION

The review led to a number of recommendations:

1. Management and funding – gaps in governance were identified, including the high turnover of core leaders at the central level, persistence of financial gaps with important donor dependency, limited MAF implementation and sub-optimal engagement of the private sector. The recommendations issued by the Programme Review on improving TB governance will guide the Pakistan NTP to further improve this core area.
2. Diagnosis – there was progress in coverage of services for TB, including diagnosis. Fragmentation of the diagnostic system across levels (from the federal to the local) and between sectors (private and public), despite improvements in modern diagnostic roll-out, hampers further progress in diagnosing more TB cases. There was inefficient use (and under-use) of molecular techniques (mostly Xpert MTB/RIF) for diagnosis, with lower rates of confirmation in the private sector. To further improve the diagnostic process coordination, integration and rationalisation should accompany the scale-up of WHO recommended rapid diagnostics, particularly in the private sector.
3. Treatment – quality treatment is not always ensured for all cases in all sectors, which could explain avoidable treatment failure and increasing resistance rates. Treatment also requires adoption of the newly recommended regimens for DS and drug-resistant TB as well as post TB lung disease management and strengthening of adherence measures taking advantage of new technologies.
4. Prevention – TPT and TB-IPC are sub-optimally implemented and need to be considerably scaled-up. The Pakistan NTP should be able to describe the TPT cascade of care and implement TB-IPC plans at both the national and the facility level.
